# Supplementary material for: Two mechanisms repress cyclin B1 translation to maintain prophase arrest in mouse oocytes
Source: Nat Commun. 2024 Nov 20;15:10044. doi: 10.1038/s41467-024-54161-w (PMC11579420; doi:10.1038/s41467-024-54161-w)
Supplement: Supplementary file 1 — Supplementary Information [file 41467_2024_54161_MOESM1_ESM.pdf]

Supplementary Materials for

Two mechanisms repress cyclin B1 translation to maintain prophase arrest in mouse oocytes

Shiya Cheng & Melina Schuh

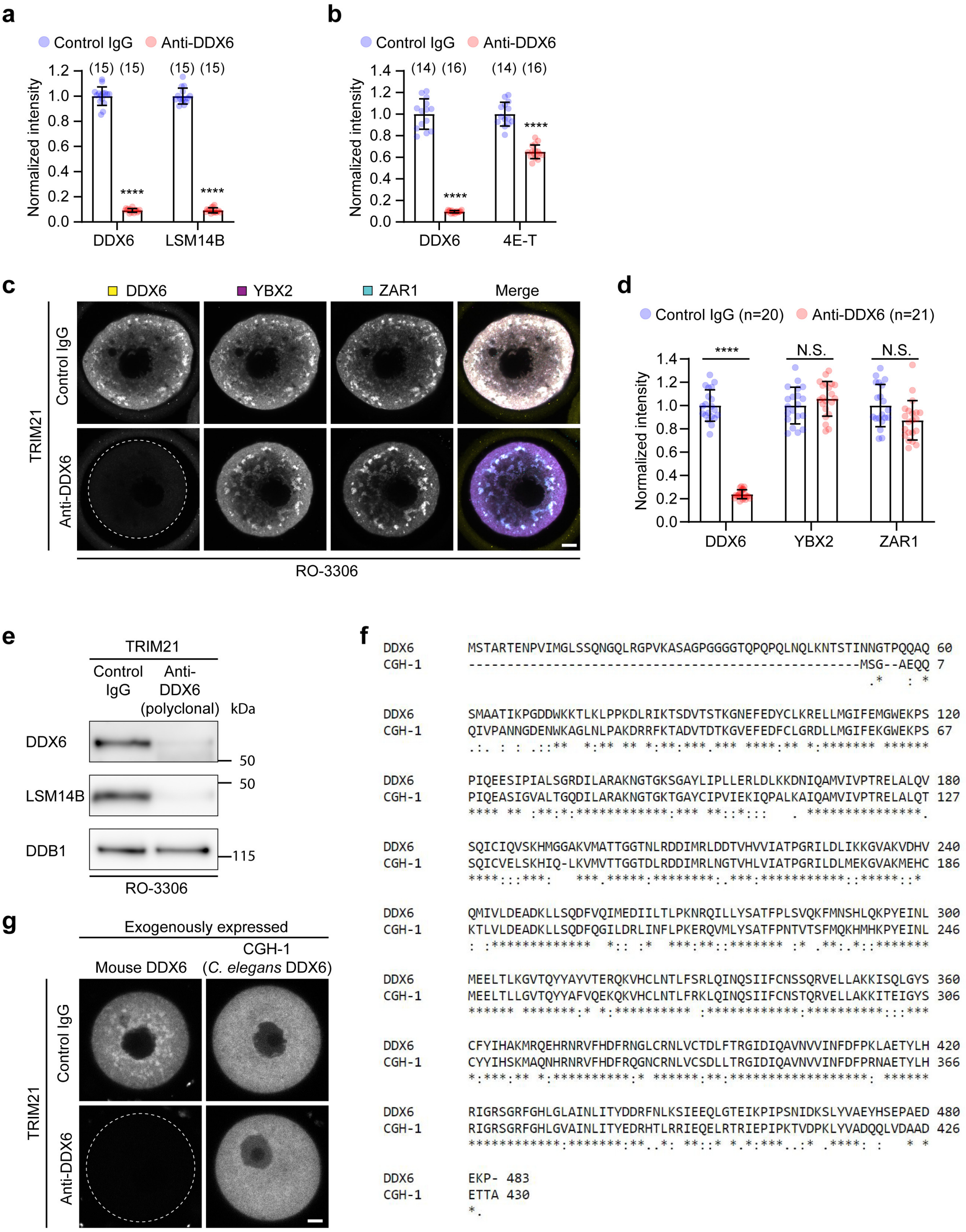
a
Control IgG
Anti-DDX6
Normalized intensity
(15) (15) (15) (15)
****
DDX6
LSM14B
b
Control IgG
Anti-DDX6
Normalized intensity
(14) (16) (14) (16)
****
DDX6
4E-T
c
DDX6
YBX2
ZAR1
Merge
TRIM21
Control IgG
Anti-DDX6
RO-3306
d
Control IgG (n=20)
Anti-DDX6 (n=21)
Normalized intensity
****
N.S.
N.S.
DDX6
YBX2
ZAR1
e
TRIM21
Control IgG
Anti-DDX6 (polyclonal)
kDa
DDX6
LSM14B
DDB1
50
50
115
RO-3306
f
DDX6 MSTARTENPVIMGLSSQNGQLRGPVKASAGPGGGGTQPQPQLNQLKNTSTINNGTPQQAQ 60
CGH-1 --MSG--AEQQ 7
DDX6 SMAATIKPGDDWKKTLKLPPKDLRIKTSDVTSTKGNEFEDYCLKRELLMGIFEMGWEKPS 120
CGH-1 QIVPANNGDENWKAGLNLPAKDRRFKTADVTDTKGVEFEDFCLGRDLLMGIFEKGWEKPS 67
DDX6 PIQEESIPIALSGRDILARAKNGTGKSGAYLIPLLERLDLKKDNIQAMVIVPTRELALQV 180
CGH-1 PIQEASIGVALTGQDILARAKNGTGKTGAYCIPVIEKIQPALKAIQAMVIVPTRELALQT 127
DDX6 SQICIQVSKHMGGAKVMATTGGTNLRDDIMRLDDTVHVVIATPGRILDLIKKGVAKVDHV 240
CGH-1 SQICVELSKHIQ-LKVMVTTGGTDLRDDIMRLNGTVHLVIATPGRILDLMEKGVAKMEHC 186
DDX6 QMIVLDEADKLLSQDFVQIMEDIILTLPKNRQILLYSATFPLSVQKFMNSHLQKPYEINL 300
CGH-1 KTLVLDEADKLLSQDFQGILDRLINFLPKERQVMLYSATFPNTVTSFMQKHMHKPYEINL 246
DDX6 MEELTLKGVTQYYAYVTERQKVHCLNTLFSRLQINQSIIFCNSSQRVELLAKKISQLGYS 360
CGH-1 MEELTLLGVTQYYAFVQEKQKVHCLNTLFRKLQINQSIIFCNSTQRVELLAKKITEIGYS 306
DDX6 CFYIHAKMRQEHRNRVFHDFRNGLCRNLVCTDLFTRGIDIQAVNVVINFDFPKLAETYLH 420
CGH-1 CYYIHSKMAQNHRNRVFHDFRQGNCRNLVCSDLLTRGIDIQAVNVVINFDFPRNAETYLH 366
DDX6 RIGRSGRFGHLGLAINLITYDDRFNLKSIEEQLGTEIKPIPSNIDKSLYVAEYHSEPAED 480
CGH-1 RIGRSGRFGHLGVAINLITYEDRHTLRRIEQELRTRIEPIPKTVDPKLYVADQQLVDAAD 426
DDX6 EKP- 483
CGH-1 ETTA 430
g
Exogenously expressed
Mouse DDX6
CGH-1 (C. elegans DDX6)
TRIM21
Control IgG
Anti-DDX6

Supplementary Figure 1: Rescue of DDX6 Trim-Away using a DDX6 homologue.

a,**b**, Quantification and normalization of the mean fluorescence intensity of DDX6, LSM14B, and 4E-T in **Fig. 1c,d**. **c**, Representative immunofluorescence images of control and DDX6-depleted oocytes. Oocytes were incubated for 6 h in the presence of 10 μM RO-3306 after injection of *Trim21* mRNA and DDX6 monoclonal antibody. Yellow, DDX6; magenta, YBX2; cyan, ZAR1. The dashed line demarcates the oocyte. **d**, Quantification and normalization of the mean fluorescence intensity of DDX6, YXB2 and ZAR1 in (**c**). **e**, Western blot analyses of mouse oocytes showing co-depletion of DDX6 and LSM14B upon DDX6 Trim-Away using an DDX6 polyclonal antibody. Oocytes were incubated for 6 h in the presence of 10 μM RO-3306 after injection of *Trim21* mRNA and the DDX6 polyclonal antibody. **f**, Protein amino acid sequence alignment of mouse DDX6 and *C. elegans* CGH-1. **g**, Exogenously expressed CGH-1 is not recognized and depleted by Trim-Away using the DDX6 monoclonal antibody. Thus, the antigen that the antibody recognizes must be in a region that differs between *C. elegans* CGH-1 and mouse DDX6. DDX6 Trim-Away was performed in mouse oocytes expressing fluorescent protein-tagged DDX6 and CGH-1. Data are all from two independent experiments. The number of analyzed oocytes is specified in italics. Data are shown as mean ± SD. *P* values were calculated using two-way ANOVA with Tukey's *post-hoc* test. *P* values are designated as $^{****}P < 0.0001$. Nonsignificant values are indicated as N.S. Scale bars, 10 μm. Source data are provided as a Source Data file.

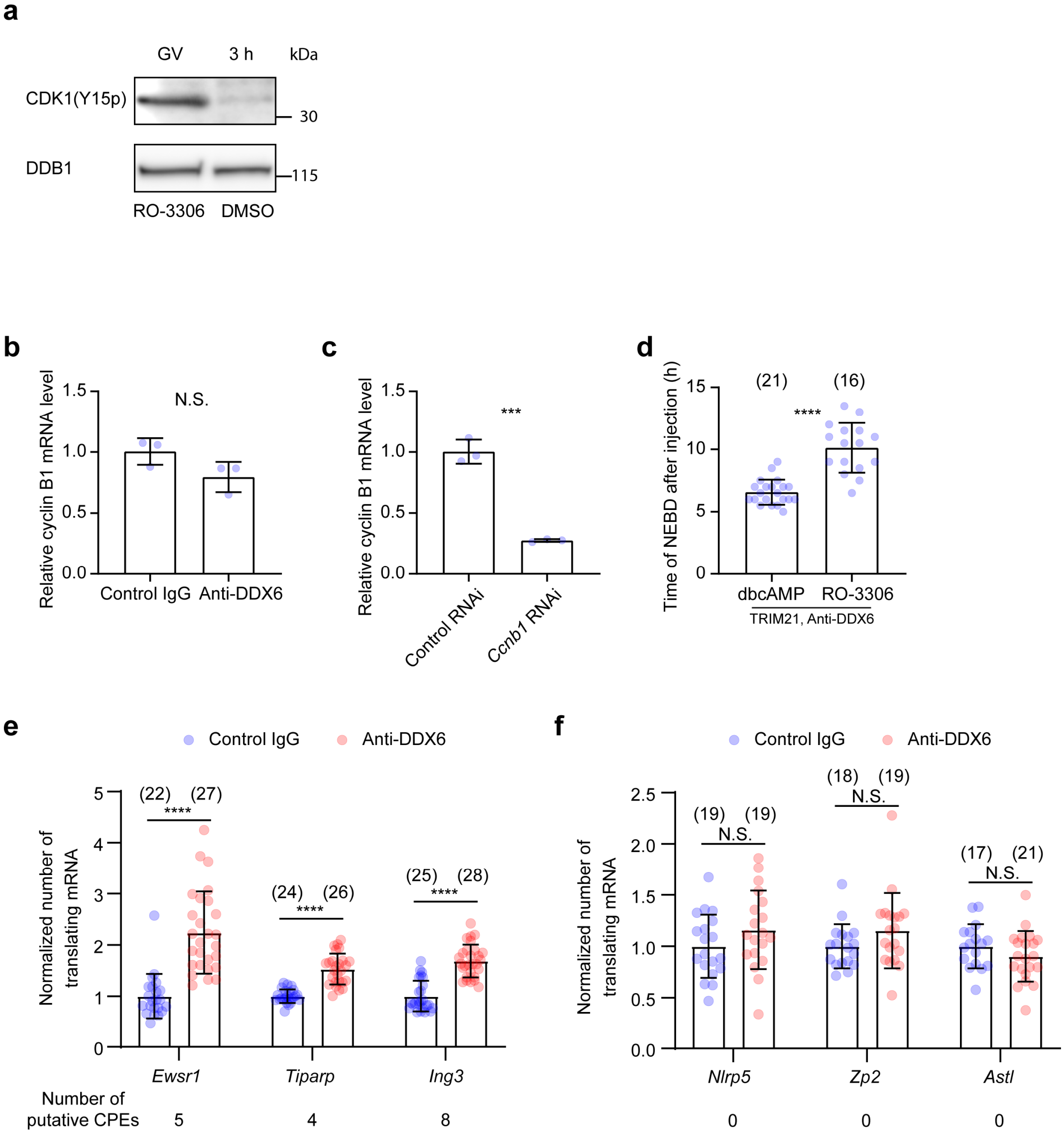
a
GV
3 h
kDa
CDK1(Y15p)
30
DDB1
115
RO-3306
DMSO
b
Relative cyclin B1 mRNA level
N.S.
Control IgG
Anti-DDX6
c
Relative cyclin B1 mRNA level
***
Control RNAi
Ccnb1 RNAi
d
Time of NEBD after injection (h)
(21)
(16)
****
dbcAMP
RO-3306
TRIM21, Anti-DDX6
e
Control IgG
Anti-DDX6
Normalized number of translating mRNA
(22) (27)
(24) (26)
(25) (28)
****
Ewsr1
Tiparp
Ing3
Number of putative CPEs
5
4
8
f
Control IgG
Anti-DDX6
Normalized number of translating mRNA
(19) (19)
(18) (19)
(17) (21)
N.S.
Nlrp5
Zp2
Astl
0
0
0

Supplementary Figure 2: DDX6 and LSM14B repress the translation of other CPEB1-bound mRNAs.

a, Western blot analyses of mouse oocytes showing phosphorylation and de-phosphorylation of CDK1 at Tyr15 before and after meiotic resumption. Oocytes were either incubated for 3 h in the presence of 10 μM RO-3306 to maintain them at the GV stage or incubated for 3 h without RO-3306 to resume meiosis. **b**, Quantitative reverse transcription PCR (RT-qPCR) results showing expression of cyclin B1 in control and DDX6/LSM14B-depleted mouse oocytes. *Actb* mRNA was used as an internal control. Oocytes were incubated for 6 h in the presence of 10 μM RO-3306 after injection of *Trim21* mRNA and DDX6 monoclonal antibody. **c**, RT-qPCR results showing expression of cyclin B1 in control and *ccnb1* RNAi oocytes. *Actb* mRNA was used as an internal control. **d**, Quantification of the time required for nuclear envelope breakdown (NEBD) after injection of *Trim21* mRNA and DDX6 monoclonal antibody. Oocytes were incubated with 250 μM dbcAMP or 10 μM RO-3306. **e**,**f**, RIBOmap assay showing the number of translating mRNAs containing multiple CPEs or no CPE[34] in control and DDX6/LSM14B-depleted mouse oocytes. The data were normalized by dividing the values of each group by the mean of the control IgG group. Oocytes were incubated for 6 h in the presence of 10 μM RO-3306 after injection of *Trim21* mRNA and DDX6 monoclonal antibody. Data are from three biological replicates (**b**, **c**) or two independent experiments (**a**, **d**–**f**). The number of analyzed oocytes is specified in italics. Data are shown as mean ± SD. *P* values were calculated using unpaired two-tailed Student's *t* test (**b**–**d**) or two-way ANOVA with Tukey's *post-hoc* test (**e**, **f**). *P* values are designated as $****P < 0.0001$, $***P < 0.001$. Nonsignificant values are indicated as N.S. Source data are provided as a Source Data file.

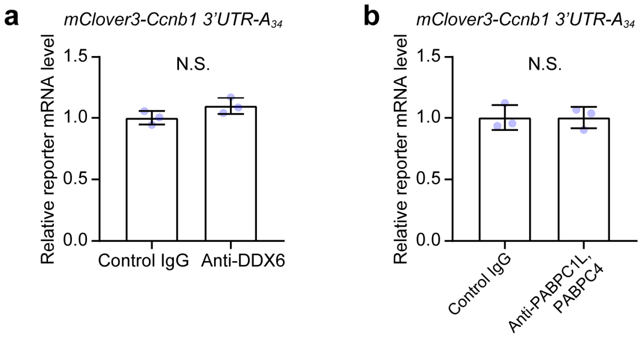
a
mClover3-Ccnb1 3'UTR-A34
Relative reporter mRNA level
1.5
1.0
0.5
0.0
N.S.
Control IgG
Anti-DDX6
b
mClover3-Ccnb1 3'UTR-A34
Relative reporter mRNA level
1.5
1.0
0.5
0.0
N.S.
Control IgG
Anti-PABPC1L, PABPC4

Supplementary Figure 3: Acute depletion of DDX6/LSM14B or PABPC1L/PABPC4 does not affect cyclin B1 reporter mRNA levels.

a,**b**, RT-qPCR results showing the relative levels of *mClove3-Ccnb1 3'UTR-*A_{34} reporter mRNA in control and DDX6/LSM14B-depleted (**a**) or PABPC1L/PABPC4-depleted (**b**) mouse oocytes. The co-injected *mScarlet* mRNA was used as an internal control. Oocytes were incubated for 8 h in the presence of 10 μM RO-3306 after injection of *Trim21* mRNA and the antibodies. Data are both from three biological replicates. Data are shown as mean ± SD. *P* values were calculated using unpaired two-tailed Student's *t* test. Nonsignificant values are indicated as N.S. Source data are provided as a Source Data file.

a
b

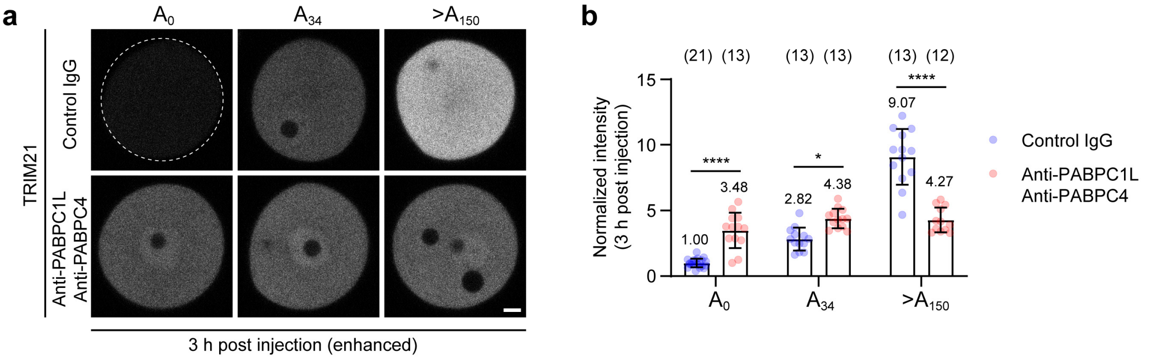

c

mRNA	*Orc6*	*Btg4*	*Tcl1b4*	···	*Actb*	···	*Sec61g*	*Tbca*
Poly(A) tail $length_{GV}$	28.40 nt	29.26 nt	29.83 nt	···	73.76 nt	···	107.13 nt	112.16 nt
Criteria	$FPKM_{GV}$ > 100, TE_{MII} / TE_{GV} > 10, Poly(A) $tail_{GV}$ < 30 nt			···	···	···	$FPKM_{GV}$ > 100, TE_{GV} > 5, Poly(A) $tail_{GV}$ > 105 nt	

d
e
f
g
h
i
j
k
l

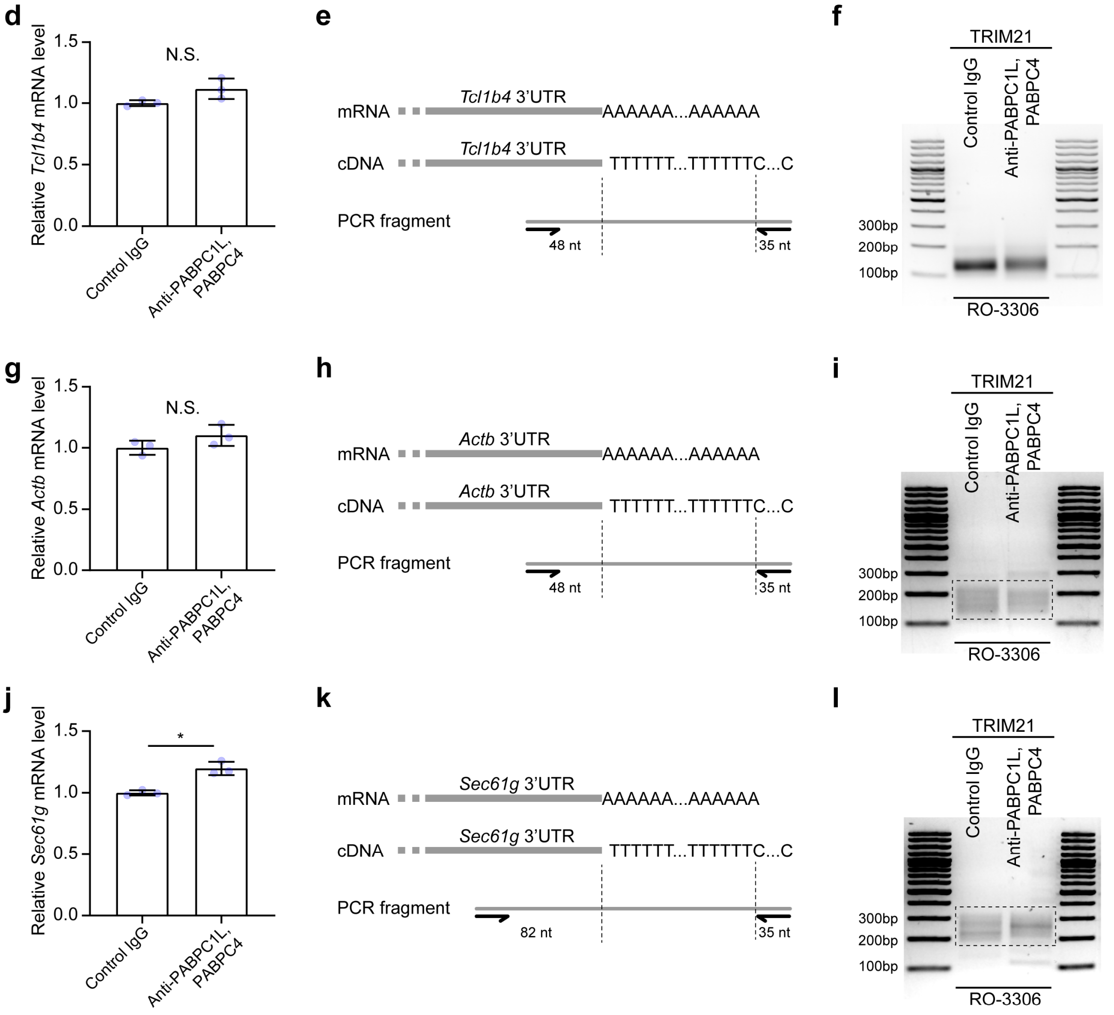

Supplementary Figure 4: Acute depletion of PABPCs does not affect mRNA stability or poly(A) tail length.

a, Representative fluorescence images of control and PABPC1L/PABPC4-depleted mouse oocytes expressing *mClove3-A*$_{0}$, *mClove3-A*$_{34}$, or *mClove3->A*$_{150}$ at 3 h after injection of these reporter mRNAs (Corresponds to **Fig. 4a**). **b**, Quantification and normalization of the mean fluorescence intensity of mClover3 in (**a**). **c**, Dormant short-tailed mRNAs that are translationally activated when oocytes resume meiosis are selected according to the criteria: $FPKM_{GV} > 100$, $TE_{MII} / TE_{GV} > 10$, Poly(A) tail $_{GV} < 30$ nt[1,2]. Actively translated long-tailed mRNAs are selected according to the criteria: $FPKM_{GV} > 100$, $TE_{GV} > 5$, Poly(A) tail $_{GV} > 105$ nt[2]. TE represents translation efficiency. **d**,**g**,**j**, RT-qPCR results showing expression of *Tcl1b4* (**d**), *Actb* (**g**) or *Sec61g* (**j**) in control and PABPC1L/PABPC4-depleted mouse oocytes. 18S rRNA was used as an internal control. Oocytes were incubated for 6 h in the presence of 10 μM RO-3306 after injection of *Trim21* mRNA and antibodies. **e**,**h**,**k**, Strategies for investigating whether acute depletion of PABPCs alters the poly(A) tail length of *Tcl1b4* (**e**), *Actb* (**h**) or *Sec61g* (**k**). **f**,**i**,**l**, Measurement of the poly(A) tail length of *Tcl1b4* (**f**), *Actb* (**i**) or *Sec61g* (**l**) in control and PABPC1L/PABPC4-depleted mouse oocytes. Oocytes were incubated for 6 h in the presence of 10 μM RO-3306 after injection of *Trim21* mRNA and antibodies. Data are from three biological replicates (**d**, **g**, **j**) or two independent experiments (**a**, **b**, **f**, **i**, **l**). The number of analyzed oocytes is specified in italics. Data are shown as mean ± SD. *P* values were calculated using two-way ANOVA with Tukey's *post-hoc* test (**b**) or unpaired two-tailed Student's *t* test (**d**, **g**, **j**). *P* values are designated as $****P < 0.0001$, $***P < 0.001$, $**P < 0.01$, $*P < 0.05$. Nonsignificant values are indicated as N.S. Scale bar, 10 μm. Source data are provided as a Source Data file.

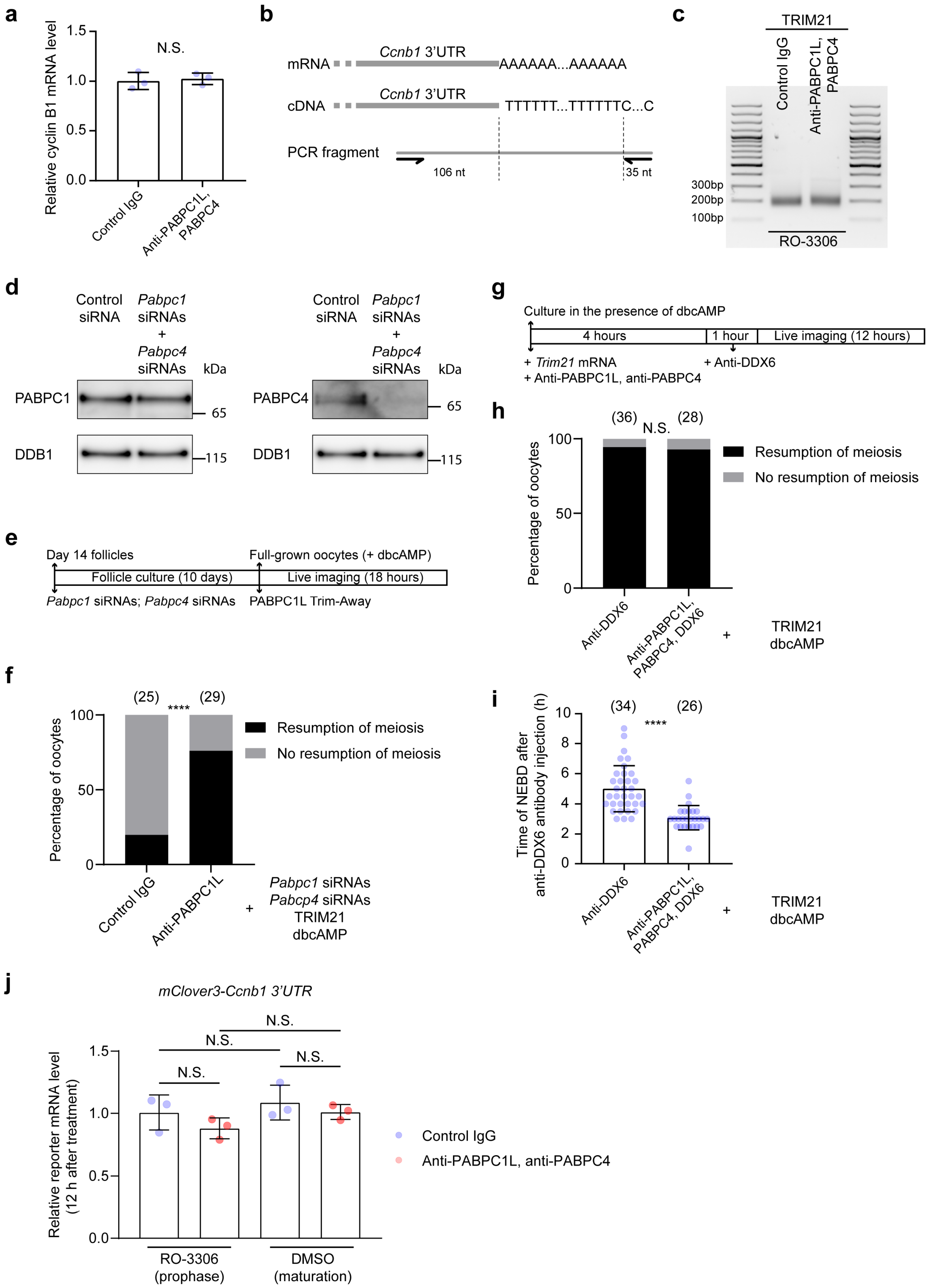
a
Relative cyclin B1 mRNA level
N.S.
Control IgG
Anti-PABPC1L, PABPC4
b
mRNA
Ccnb1 3'UTR
AAAAAA...AAAAAA
cDNA
TTTTTT...TTTTTTC...C
PCR fragment
106 nt
35 nt
c
TRIM21
Control IgG
Anti-PABPC1L, PABPC4
300bp
200bp
100bp
RO-3306
d
Control siRNA
Pabpc1 siRNAs + Pabpc4 siRNAs
kDa
PABPC1
DDB1
PABPC4
65
115
e
Day 14 follicles
Follicle culture (10 days)
Pabpc1 siRNAs; Pabpc4 siRNAs
Full-grown oocytes (+ dbcAMP)
Live imaging (18 hours)
PABPC1L Trim-Away
f
Percentage of oocytes
(25)
****
(29)
Resumption of meiosis
No resumption of meiosis
Control IgG
Anti-PABPC1L
+
Pabpc1 siRNAs
Pabcp4 siRNAs
TRIM21
dbcAMP
g
Culture in the presence of dbcAMP
4 hours
1 hour
Live imaging (12 hours)
+ Trim21 mRNA
+ Anti-PABPC1L, anti-PABPC4
+ Anti-DDX6
h
Percentage of oocytes
(36)
N.S.
(28)
Resumption of meiosis
No resumption of meiosis
Anti-DDX6
Anti-PABPC1L, PABPC4, DDX6
+
TRIM21
dbcAMP
i
Time of NEBD after anti-DDX6 antibody injection (h)
(34)
****
(26)
Anti-DDX6
Anti-PABPC1L, PABPC4, DDX6
+
TRIM21
dbcAMP
j
mClover3-Ccnb1 3'UTR
Relative reporter mRNA level (12 h after treatment)
N.S.
N.S.
N.S.
N.S.
Control IgG
Anti-PABPC1L, anti-PABPC4
RO-3306 (prophase)
DMSO (maturation)

Supplementary Figure 5: PABPCs are essential for maintaining prophase arrest.

a, RT-qPCR results showing expression of cyclin B1 in control and PABPC1L/PABPC4-depleted mouse oocytes. 18S rRNA was used as an internal control. Oocytes were incubated for 6 h in the presence of 10 μM RO-3306 after injection of *Trim21* mRNA and antibodies. **b**, Strategy for investigating whether acute depletion of PABPCs alters the poly(A) tail length of cyclin B1 mRNA. **c**, Measurement of the poly(A) tail length of cyclin B1 mRNA in control and PABPC1L/PABPC4-depleted mouse oocytes. Oocytes were incubated for 6 h in the presence of 10 μM RO-3306 after injection of *Trim21* mRNA and antibodies. **d**, Western blot analyses showing that PABPC4, but not PABPC1, was successfully knocked down by RNAi. **e**, Schematic diagram of the experiment in (**f**). *Pabpc1* RNAi and *Pabpc4* RNAi was performed, followed by acute depletion of PABPC1L. **f**, Percentage of control and PABPC1L-depleted mouse oocytes that resume meiosis in 18 h in the presence of dbcAMP. *Pabpc1* RNAi and *Pabpc4* RNAi was performed in all oocytes, but only PABPC4 was successfully knocked down in protein level (**d**). **g**, Schematic diagram of the experiment in (**h** and **i**). Acute depletion of PABPC1L and PABPC4 was performed, followed by acute depletion of DDX6 and LSM14B. Oocytes were incubated and imaged in the medium containing dbcAMP. **h**, Percentage of mouse oocytes that resume meiosis in 12 h in the presence of dbcAMP. **i**, Quantification of the time required for NEBD after injection of DDX6 monoclonal antibody. Only oocytes that resume meiosis in 12 h were quantified. **j**, RT-qPCR results showing the relative levels of *mClove3-Ccnb1 3'UTR* reporter mRNA in control and PABPC1L/PABPC4-depleted mouse oocytes (Corresponds to **Fig. 5d**). *Actb* mRNA was used as an internal control. Oocytes were incubated for 12 h in the presence or absence of 10 μM RO-3306 after injection of the reporter mRNA and one hour recovery. Data are from three biological replicates (**a**, **j**) or two independent experiments (**b**–**i**). The number of analyzed oocytes is specified in italics. Data are shown as mean ± SD. P values were calculated using unpaired two-tailed Student's t test (**a**, **i**), one-way ANOVA with Tukey's *post-hoc* test (**j**) or two-tailed Fisher's exact test (**f**, **h**). P values are designated as $^{****}P < 0.0001$. Nonsignificant values are indicated as N.S. Source data are provided as a Source Data file.

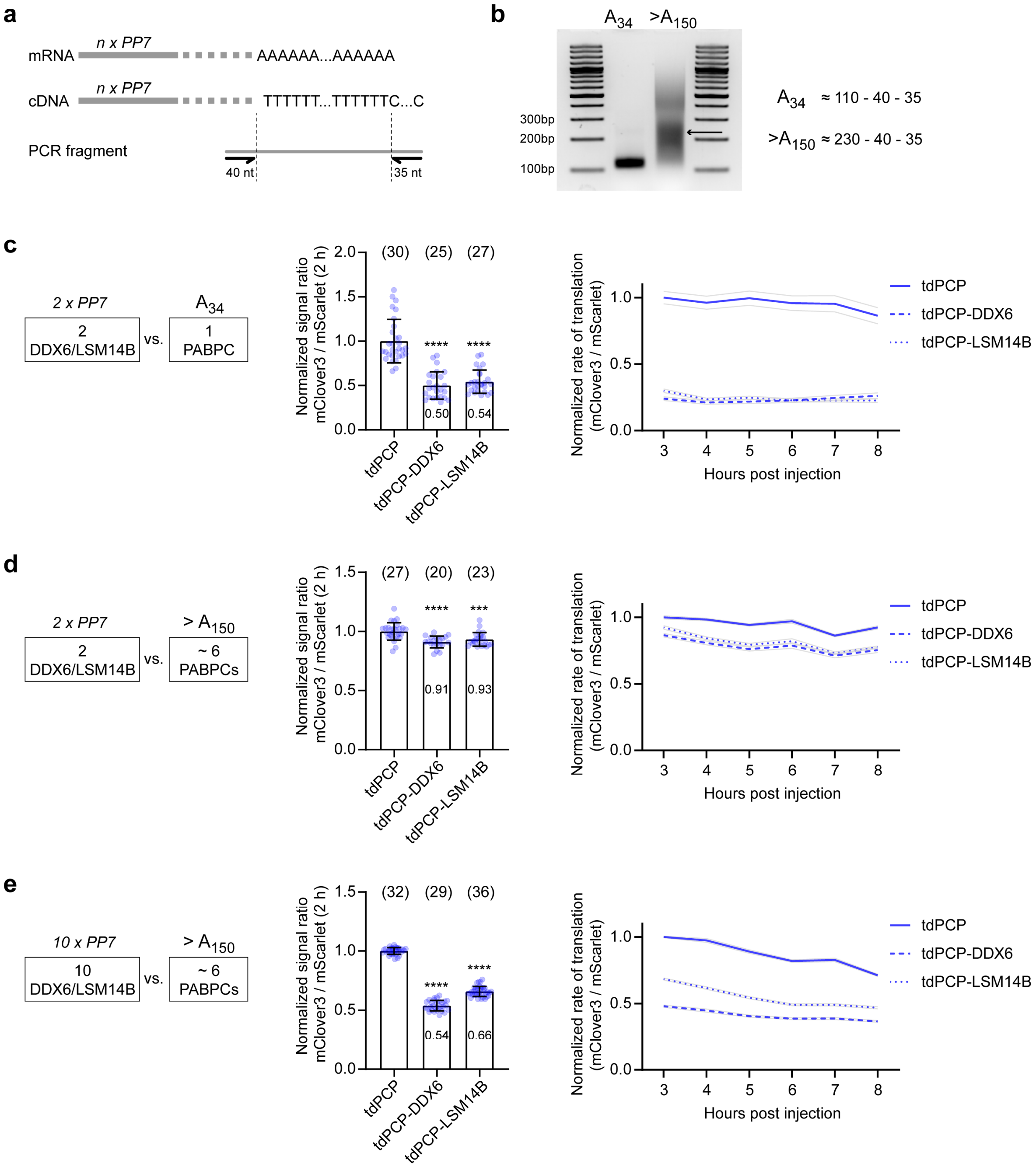
a
mRNA
n x PP7
AAAAAA...AAAAAA
cDNA
n x PP7
TTTTTT...TTTTTTC...C
PCR fragment
40 nt
35 nt
b
A34
>A150
300bp
200bp
100bp
A34 ≈ 110 - 40 - 35
>A150 ≈ 230 - 40 - 35
c
2 x PP7
A34
2 DDX6/LSM14B
vs.
1 PABPC
Normalized signal ratio mClover3 / mScarlet (2 h)
(30) (25) (27)
0.50
0.54
tdPCP
tdPCP-DDX6
tdPCP-LSM14B
Normalized rate of translation (mClover3 / mScarlet)
Hours post injection
tdPCP
tdPCP-DDX6
tdPCP-LSM14B
d
2 x PP7
> A150
2 DDX6/LSM14B
vs.
~ 6 PABPCs
(27) (20) (23)
0.91
0.93
e
10 x PP7
> A150
10 DDX6/LSM14B
vs.
~ 6 PABPCs
(32) (29) (36)
0.54
0.66

Supplementary Figure 6: PABPCs and DDX6/LSM14B counteract each other on the same mRNA.

a, Strategy for measuring the poly(A) tail length of *mClover3-2xPP7-A34* and *mClover3-2xPP7->A150* reporter mRNAs in vitro. **b**, Measurement of the poly(A) tail length of *mClover3-2xPP7-A34* and *mClover3-2xPP7->A150* reporter mRNAs in vitro. **c–e**, The reporter mRNA *mClover3* with different numbers of PP7s and different lengths of poly(A) tails, the injection control *mScarlet*, and *tdPCP*, *tdPCP-Ddx6* or *tdPCP-Lsm14b* were co-injected into GV oocytes (Corresponds to **Fig. 6**). The fluorescence intensity ratio of mClover3 to mScarlet was quantified and normalized 2 h after mRNA injection. The rate of translation over time was quantified by dividing the increase in mClover3 signal per hour by the increase in mScarlet signal per hour. The first data of the *tdPCP* group was normalized to "1". Data are all from two independent experiments. The number of analyzed oocytes is specified in italics. Data are shown as mean ± SD (bar charts) or mean ± SEM (curves). *P* values were calculated using one-way ANOVA with Tukey's *post-hoc* test. *P* values are designated as $****P < 0.0001$, $***P < 0.001$. Source data are provided as a Source Data file.

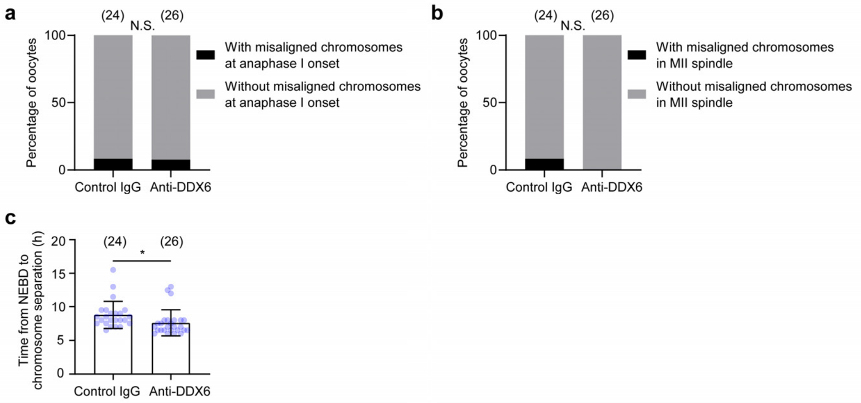
a
(24)
N.S.
(26)
Percentage of oocytes
100
50
0
Control IgG
Anti-DDX6
With misaligned chromosomes at anaphase I onset
Without misaligned chromosomes at anaphase I onset
b
(24)
N.S.
(26)
Percentage of oocytes
100
50
0
Control IgG
Anti-DDX6
With misaligned chromosomes in MII spindle
Without misaligned chromosomes in MII spindle
c
(24)
*
(26)
Time from NEBD to chromosome separation (h)
20
15
10
5
0
Control IgG
Anti-DDX6

Supplementary Figure 7: Acute depletion of DDX6 and LSM14B does not affect spindle assembly and chromosome alignment.

a, Quantification of chromosome misalignment at anaphase I onset in control and DDX6/LSM14B-depleted mouse oocytes (Corresponds to **Fig. 7a**). **b**, Quantification of chromosome misalignment in MII spindle in control and DDX6/LSM14B-depleted mouse oocytes (Corresponds to **Fig. 7a**). **c**, Quantification of the time from NEBD to chromosome separation in control and DDX6/LSM14B-depleted mouse oocytes (Corresponds to **Fig. 7a**). Data are from two independent experiments. The number of analyzed oocytes is specified in italics. Data are shown as mean ± SD (**c**). *P* values were calculated using two-tailed Fisher's exact test (**a** and **b**) or unpaired two-tailed Student's *t* test (**c**). *P* values are designated as $*P < 0.05$. Nonsignificant values are indicated as N.S. Source data are provided as a Source Data file.

References

1. Luong, X.G., Daldello, E.M., Rajkovic, G., Yang, C.R. & Conti, M. Genome-wide analysis reveals a switch in the translational program upon oocyte meiotic resumption. *Nucleic Acids Research* **48**, 3257-3276 (2020).

2. Xiong, Z.Q. *et al.* Ultrasensitive Ribo-seq reveals translational landscapes during mammalian oocyte-to-embryo transition and pre-implantation development. *Nat Cell Biol* **24**, 968-+ (2022).
