# Supplementary material for: Two mechanisms repress cyclin B1 translation to maintain prophase arrest in mouse oocytes
Source: Nat Commun. 2024 Nov 20;15:10044. doi: 10.1038/s41467-024-54161-w (PMC11579420; doi:10.1038/s41467-024-54161-w)
Supplement: Supplementary file 8 — Reporting Summary [file 41467_2024_54161_MOESM8_ESM.pdf]

nature portfolio

Corresponding author(s): Melina Schuh

Last updated by author(s): Jan 24, 2024

Reporting Summary

Nature Portfolio wishes to improve the reproducibility of the work that we publish. This form provides structure for consistency and transparency in reporting. For further information on Nature Portfolio policies, see our Editorial Policies and the Editorial Policy Checklist.

Statistics

For all statistical analyses, confirm that the following items are present in the figure legend, table legend, main text, or Methods section.

n/a	Confirmed	
☐	☒	The exact sample size (*n*) for each experimental group/condition, given as a discrete number and unit of measurement
☐	☒	A statement on whether measurements were taken from distinct samples or whether the same sample was measured repeatedly
☐	☒	The statistical test(s) used AND whether they are one- or two-sided<br>*Only common tests should be described solely by name; describe more complex techniques in the Methods section.*
☒	☐	A description of all covariates tested
☒	☐	A description of any assumptions or corrections, such as tests of normality and adjustment for multiple comparisons
☐	☒	A full description of the statistical parameters including central tendency (e.g. means) or other basic estimates (e.g. regression coefficient) AND variation (e.g. standard deviation) or associated estimates of uncertainty (e.g. confidence intervals)
☒	☐	For null hypothesis testing, the test statistic (e.g. *F*, *t*, *r*) with confidence intervals, effect sizes, degrees of freedom and *P* value noted<br>*Give P values as exact values whenever suitable.*
☒	☐	For Bayesian analysis, information on the choice of priors and Markov chain Monte Carlo settings
☒	☐	For hierarchical and complex designs, identification of the appropriate level for tests and full reporting of outcomes
☒	☐	Estimates of effect sizes (e.g. Cohen's *d*, Pearson's *r*), indicating how they were calculated

Our web collection on statistics for biologists contains articles on many of the points above.

Software and code

Policy information about availability of computer code

Data collection	ZEN 2.1 (black edition)
Data analysis	Fiji ImageJ, Imaris 9.3, ZEN 2 (blue edition), Excel 2016, GraphPad Prism 9.1.2

For manuscripts utilizing custom algorithms or software that are central to the research but not yet described in published literature, software must be made available to editors and reviewers. We strongly encourage code deposition in a community repository (e.g. GitHub). See the Nature Portfolio guidelines for submitting code & software for further information.

Data

Policy information about availability of data

All manuscripts must include a data availability statement. This statement should provide the following information, where applicable:

- Accession codes, unique identifiers, or web links for publicly available datasets
- A description of any restrictions on data availability
- For clinical datasets or third party data, please ensure that the statement adheres to our policy

All relevant and raw data supporting the finding of this study are available from the corresponding author on reasonable request. The primary microscopy data were not uploaded to a data repository due to their large size, but are available from the corresponding author on reasonable request.

Research involving human participants, their data, or biological material

Policy information about studies with human participants or human data. See also policy information about sex, gender (identity/presentation), and sexual orientation and race, ethnicity and racism.

Reporting on sex and gender	*Use the terms sex (biological attribute) and gender (shaped by social and cultural circumstances) carefully in order to avoid confusing both terms. Indicate if findings apply to only one sex or gender; describe whether sex and gender were considered in study design; whether sex and/or gender was determined based on self-reporting or assigned and methods used. Provide in the source data disaggregated sex and gender data, where this information has been collected, and if consent has been obtained for sharing of individual-level data; provide overall numbers in this Reporting Summary. Please state if this information has not been collected. Report sex- and gender-based analyses where performed, justify reasons for lack of sex- and gender-based analysis.*
Reporting on race, ethnicity, or other socially relevant groupings	*Please specify the socially constructed or socially relevant categorization variable(s) used in your manuscript and explain why they were used. Please note that such variables should not be used as proxies for other socially constructed/relevant variables (for example, race or ethnicity should not be used as a proxy for socioeconomic status). Provide clear definitions of the relevant terms used, how they were provided (by the participants/respondents, the researchers, or third parties), and the method(s) used to classify people into the different categories (e.g. self-report, census or administrative data, social media data, etc.) Please provide details about how you controlled for confounding variables in your analyses.*
Population characteristics	*Describe the covariate-relevant population characteristics of the human research participants (e.g. age, genotypic information, past and current diagnosis and treatment categories). If you filled out the behavioural & social sciences study design questions and have nothing to add here, write "See above."*
Recruitment	*Describe how participants were recruited. Outline any potential self-selection bias or other biases that may be present and how these are likely to impact results.*
Ethics oversight	*Identify the organization(s) that approved the study protocol.*

Note that full information on the approval of the study protocol must also be provided in the manuscript.

Field-specific reporting

Please select the one below that is the best fit for your research. If you are not sure, read the appropriate sections before making your selection.

☒ Life sciences ☐ Behavioural & social sciences ☐ Ecological, evolutionary & environmental sciences

For a reference copy of the document with all sections, see nature.com/documents/nr-reporting-summary-flat.pdf

Life sciences study design

All studies must disclose on these points even when the disclosure is negative.

Sample size	No statistical methods were used to predetermine sample size. In retrospective, achieved sample sizes were determined to be adequate based on the magnitude and consistency of measurable differences between groups. Most importantly, sample size per experiment was dictated by the number of oocytes that could be processed (microinjection and live imaging) within a reasonable time by the researcher without affecting oocyte quality.
Data exclusions	No data were excluded from the analysis.
Replication	All data are from at least two independent experiments / multiple biological replicates. All attempts at replication were successful.
Randomization	In most experiments (except for RT-qPCR), mouse oocytes were collected from multiple ovaries/animals and then pooled. They were then randomly assigned to different experimental groups.
Blinding	Investigators were not blinded to allocation during experiments and outcome assessment, as each experiment was performed by one researcher alone. Thus, blinding during group allocation was not possible to ensure samples received the right treatment/manipulation during experiment. Blinding was also not possible during data analysis, as data analysis was performed by the same researcher that conducted the experiment.

Reporting for specific materials, systems and methods

We require information from authors about some types of materials, experimental systems and methods used in many studies. Here, indicate whether each material, system or method listed is relevant to your study. If you are not sure if a list item applies to your research, read the appropriate section before selecting a response.

Materials & experimental systems

n/a	Involved in the study
☐	☒ Antibodies
☒	☐ Eukaryotic cell lines
☒	☐ Palaeontology and archaeology
☐	☒ Animals and other organisms
☒	☐ Clinical data
☒	☐ Dual use research of concern
☒	☐ Plants

Methods

n/a	Involved in the study
☒	☐ ChIP-seq
☒	☐ Flow cytometry
☒	☐ MRI-based neuroimaging

Antibodies

Antibodies used

Primary antibodies: mouse anti-DDX6 (Sigma-Aldrich #SAB4200837), rabbit anti-LSM14B (Thermo Fisher Scientific #PA5-66371), rabbit anti-4E-T (Thermo Fisher Scientific #PA5-51680), rabbit anti-YBX2 (Abcam #ab33164), guinea pig anti-ZAR1 (made by Cambridge Research Biochemicals), mouse anti-CDK1 (Thermo Fisher Scientific #MA5-11472), rabbit anti-phospho-CDK1(Tyr15) (Cell Signaling Technology #9111), rabbit anti-DDX6 (Thermo Fisher Scientific #A300-461A), rabbit anti-CPEB1 (Proteintech #13274-1-AP), mouse anti-PABPC1L (Santa Cruz Biotechnology #sc-515476), rabbit anti-PABPC4 (Thermo Fisher Scientific #PA5-96483), rabbit anti-DDX6 (Abcam #ab174277), rabbit anti-DDB1 (Abcam #ab109027), mouse anti-cyclin B1 (Santa Cruz Biotechnology #sc-245), rabbit anti-MOS (Santa Cruz Biotechnology #sc-1093-R), rabbit anti-PABPC1 (Proteintech #10970-1-AP).
Secondary antibodies: Alexa Fluor 488-conjugated goat anti-mouse IgG highly cross-adsorbed secondary antibody (Thermo Fisher Scientific #A-11029), Alexa Fluor 568-conjugated goat anti-rabbit IgG cross-adsorbed secondary antibody (Thermo Fisher Scientific #A-11011), Alexa Fluor 647-conjugated goat anti-guinea pig IgG highly cross-adsorbed secondary antibody (Thermo Fisher Scientific #A-21450), HRP-conjugated goat anti-mouse IgG (Abcam #ab205719), HRP-conjugated goat anti-rabbit IgG (Abcam #ab205718).

Validation

Guinea pig anti-ZAR1 (made by Cambridge Research Biochemicals) was validated in a previous study (PMID: 36264786). All other used antibodies were validated commercially. Certificates of analysis for the approved applications by the manufacturer and references are available on the company websites.

Animals and other research organisms

Policy information about studies involving animals; ARRIVE guidelines recommended for reporting animal research, and Sex and Gender in Research

Laboratory animals

7- to 10-week-old mice, C57BL/6N and CD1

Wild animals

No wild animals were used in this study.

Reporting on sex

This study investigates the development of female reproductive cells called oocytes. We only used ovaries from female mice, but no materials from males.

Field-collected samples

No field-collected samples were used in this study.

Ethics oversight

Maintenance and handling of all mice were carried out in the animal facility of Max Planck Institute for Multidisciplinary Sciences according to international animal welfare rules (Federation for Laboratory Animal Science Associations guidelines and recommendations). Requirements of formal control of the German national authorities and funding organizations were satisfied, and the study received approval by the Niedersächsisches Landesamt für Verbraucherschutz und Lebensmittelsicherheit (LAVES).

Note that full information on the approval of the study protocol must also be provided in the manuscript.

Plants

Seed stocks

Report on the source of all seed stocks or other plant material used. If applicable, state the seed stock centre and catalogue number. If plant specimens were collected from the field, describe the collection location, date and sampling procedures.

Novel plant genotypes

Describe the methods by which all novel plant genotypes were produced. This includes those generated by transgenic approaches, gene editing, chemical/radiation-based mutagenesis and hybridization. For transgenic lines, describe the transformation method, the number of independent lines analyzed and the generation upon which experiments were performed. For gene-edited lines, describe the editor used, the endogenous sequence targeted for editing, the targeting guide RNA sequence (if applicable) and how the editor was applied.

Authentication

Describe any authentication procedures for each seed stock used or novel genotype generated. Describe any experiments used to assess the effect of a mutation and, where applicable, how potential secondary effects (e.g. second site T-DNA insertions, mosiacism, off-target gene editing) were examined.
